# 1-(3,5-Di­meth­oxy­phen­yl)-4,5-dimethyl-2-phenyl-1*H*-imidazole

**DOI:** 10.1107/S1600536813023684

**Published:** 2013-09-04

**Authors:** G. Divya, K. Saravanan, S. Santhiya, K. Chandralekha, S. Lakshmi

**Affiliations:** a Research Department of Physics, SDNB Vaishnav College for Women, Chennai 600 044, India; b Department of Chemistry, Easwari Engineering College, Chennai, India

## Abstract

In the title mol­ecule, C_19_H_20_N_2_O_2_, the imidazole ring makes dihedral angles of 57.29 (5) and 31.54 (5)° with the attached di­meth­oxy­phenyl residue and the phenyl ring, respectively. The dihedral angle between the di­meth­oxy­phenyl and phenyl rings is 61.15 (5)°. In the crystal, pairs of C—H⋯N hydrogen bonds connect the mol­ecules into inversion dimers.

## Related literature

For the pharmacological activity of imidazole derivatives, see: Zala *et al.* (2012 ▶). For imidazole derivatives as ligands for Ir^3+^ complexes, see: Saravanan *et al.* (2011 ▶); Gayathri *et al.* (2010 ▶).
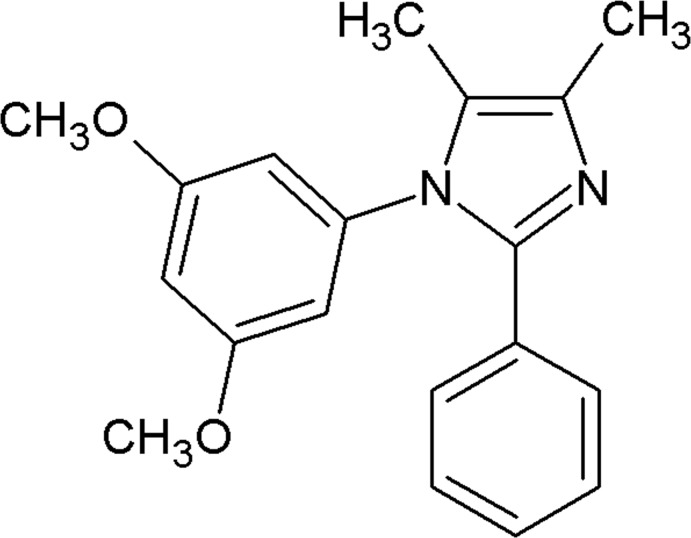


## Experimental

### 

#### Crystal data

C_19_H_20_N_2_O_2_
*M* *_r_* = 308.37
Triclinic, 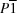

*a* = 8.363 (5) Å
*b* = 10.267 (5) Å
*c* = 10.481 (5) Å
α = 75.043 (5)°
β = 75.789 (5)°
γ = 74.576 (5)°
*V* = 822.9 (7) Å^3^
*Z* = 2
Mo *K*α radiation
μ = 0.08 mm^−1^
*T* = 295 K
0.35 × 0.25 × 0.20 mm

#### Data collection

Bruker Kappa APEXII CCD diffractometer
Absorption correction: multi-scan (*SADABS*; Bruker, 2004 ▶) *T*_min_ = 0.912, *T*_max_ = 0.984
16534 measured reflections
3780 independent reflections
2991 reflections with *I* > 2σ(*I*)
*R*_int_ = 0.028

#### Refinement

*R*[*F* ^2^ > 2σ(*F* ^2^)] = 0.042
*wR*(*F* ^2^) = 0.126
*S* = 1.03
3780 reflections
213 parameters
H-atom parameters constrained
Δρ_max_ = 0.22 e Å^−3^
Δρ_min_ = −0.17 e Å^−3^

### 

Data collection: *APEX2* (Bruker, 2004 ▶); cell refinement: *APEX2* and *SAINT* (Bruker, 2004 ▶); data reduction: *SAINT* and *XPREP* (Bruker, 2004 ▶); program(s) used to solve structure: *SIR92* (Altomare *et al.*, 1993 ▶); program(s) used to refine structure: *SHELXL97* (Sheldrick, 2008 ▶); molecular graphics: *ORTEP-3 for Windows* (Farrugia, 2012 ▶); software used to prepare material for publication: *PLATON* (Spek, 2009 ▶).

## Supplementary Material

Crystal structure: contains datablock(s) I, New_Global_Publ_Block. DOI: 10.1107/S1600536813023684/bt6926sup1.cif

Structure factors: contains datablock(s) I. DOI: 10.1107/S1600536813023684/bt6926Isup2.hkl

Supplementary material file. DOI: 10.1107/S1600536813023684/bt6926Isup3.cml

Additional supplementary materials:  crystallographic information; 3D view; checkCIF report

## Figures and Tables

**Table 1:** Hydrogen-bond geometry (Å, °)

*D*—H⋯*A*	*D*—H	H⋯*A*	*D*⋯*A*	*D*—H⋯*A*
C15—H15⋯N1^i^	0.93	2.62	3.516 (2)	162

Symmetry code: (i) 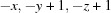.

**Fig. 1.**
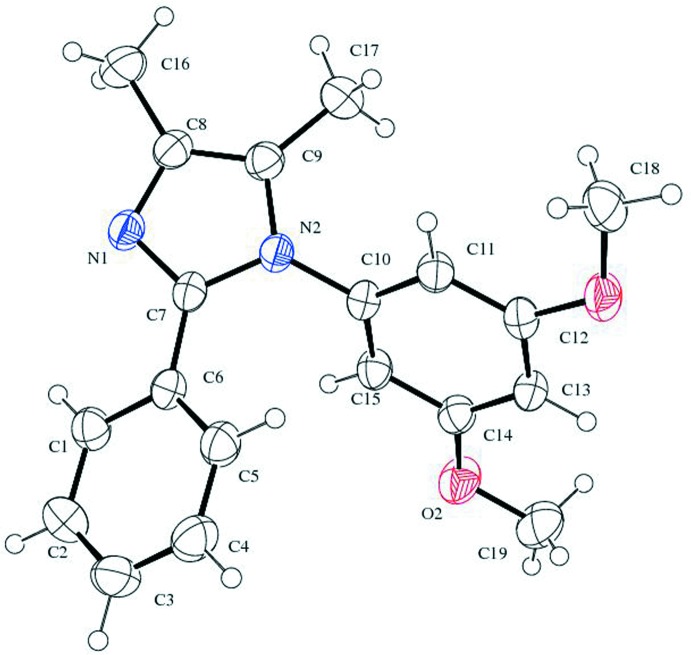
The molecular structure of the title compound,with displacement ellipsoids drawn at the 50% probability level·H atoms are shown as small sphere of arbitrary radius.

**Fig. 2.**
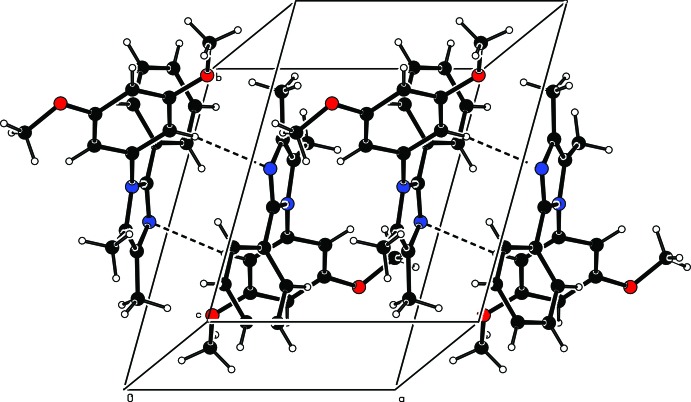
The packing of the title compound,viewed down the c axis; dashed lines indicates hydrogen bonds.

## supplementary crystallographic information

### 1. Comment

The title compound was synthesized in an attempt to develop highly sensitive chemosensors for transition metal ions. Similar compounds have the potential to be used as a ligand for synthesizing Ir^3+^ complexes (Saravanan *et al.*, 2011; Gayathri *et al.*, 2010). Imidazole derivatives are found to have diverse activities like anti-inflammatory and antimicrobial activity (Zala *et al.*, 2012).

In the title molecule (Fig.1), the imidazole ring makes dihedral angles of 57.29 (5)° and 31.54 (5)° with the attached dimethoxyphenyl residue and the phenyl ring, respectively. The dihedral angle between the benzene and phenyl rings is 61.15 (5)°. Two inversion related molecules (Table 1) connected by C–H···N hydrogen bonds form a centrosymmetric dimer (Fig. 2).

### 2. Experimental

To pure biacetyl (1.48 g, 15 mmol) in ethanol (10 ml), 3,5 dimethoxyaniline (2.30 g,15 mmol), ammonium acetate(7.0 g, 15 mmol) and benzaldehyde (1.5 g 15 mmol) were added for a period of about one hour by maintaining the temperature at 333 K. The reaction mixture was refluxed for five days and extracted with dichloromethane. The solid which separated was purified by column chromatography using hexane:ethyl acetate as the eluent. Yield: 2.1 g (45%).

### 3. Refinement

The positions of all the hydrogen atoms were identified from a difference electron density map. Nevertheless, hydrogen atoms were placed in calculated positions with distances C—H ranging from 0.93 to 0.96 Å and refined using a riding model with *U*_iso_(H) = 1.5 *U*_eq_(C) for methyl groups and *U*_iso_(H) = 1.2 *U*_eq_(C) for the remaining H atoms.

### Crystal data

**Table:**

C_19_H_20_N_2_O_2_	*Z* = 2
*M**_r_* = 308.37	*F*(000) = 328
Triclinic, *P*1	*D*_x_ = 1.245 Mg m^−^^3^
Hall symbol: -P 1	Melting point: 396.15 K
*a* = 8.363 (5) Å	Mo *K*α radiation, λ = 0.71073 Å
*b* = 10.267 (5) Å	Cell parameters from 7500 reflections
*c* = 10.481 (5) Å	θ = 2.1–31.4°
α = 75.043 (5)°	µ = 0.08 mm^−^^1^
β = 75.789 (5)°	*T* = 295 K
γ = 74.576 (5)°	Block, colourless
*V* = 822.9 (7) Å^3^	0.35 × 0.25 × 0.20 mm

### Data collection

**Table:**

Bruker Kappa APEXII CCD diffractometer	3780 independent reflections
Radiation source: fine-focus sealed tube	2991 reflections with *I* > 2σ(*I*)
Graphite monochromator	*R*_int_ = 0.028
ω and φ scan	θ_max_ = 27.5°, θ_min_ = 2.1°
Absorption correction: multi-scan (*SADABS*; Bruker, 2004)	*h* = −10→10
*T*_min_ = 0.912, *T*_max_ = 0.984	*k* = −12→13
16534 measured reflections	*l* = −13→13

### Refinement

**Table:**

Refinement on *F*^2^	Secondary atom site location: difference Fourier map
Least-squares matrix: full	Hydrogen site location: inferred from neighbouring sites
*R*[*F*^2^ > 2σ(*F*^2^)] = 0.042	H-atom parameters constrained
*wR*(*F*^2^) = 0.126	*w* = 1/[σ^2^(*F*_o_^2^) + (0.0645*P*)^2^ + 0.1449*P*] where *P* = (*F*_o_^2^ + 2*F*_c_^2^)/3
*S* = 1.03	(Δ/σ)_max_ < 0.001
3780 reflections	Δρ_max_ = 0.22 e Å^−^^3^
213 parameters	Δρ_min_ = −0.17 e Å^−^^3^
0 restraints	Extinction correction: *SHELXL97* (Sheldrick, 2008), Fc^*^=kFc[1+0.001xFc^2^λ^3^/sin(2θ)]^-1/4^
Primary atom site location: structure-invariant direct methods	Extinction coefficient: 0.041 (5)

### Special details

**Table:**

Geometry. All e.s.d.'s (except the e.s.d. in the dihedral angle between two l.s. planes) are estimated using the full covariance matrix. The cell e.s.d.'s are taken into account individually in the estimation of e.s.d.'s in distances, angles and torsion angles; correlations between e.s.d.'s in cell parameters are only used when they are defined by crystal symmetry. An approximate (isotropic) treatment of cell e.s.d.'s is used for estimating e.s.d.'s involving l.s. planes.
Refinement. Refinement of *F*^2^ against ALL reflections. The weighted *R*-factor *wR* and goodness of fit *S* are based on *F*^2^, conventional *R*-factors *R* are based on *F*, with *F* set to zero for negative *F*^2^. The threshold expression of *F*^2^ > σ(*F*^2^) is used only for calculating *R*-factors(gt) *etc*. and is not relevant to the choice of reflections for refinement. *R*-factors based on *F*^2^ are statistically about twice as large as those based on *F*, and *R*- factors based on ALL data will be even larger.

### Fractional atomic coordinates and isotropic or equivalent isotropic displacement parameters (Å^2^)

**Table:**

	*x*	*y*	*z*	*U*_iso_*/*U*_eq_	
C1	0.08801 (17)	0.29240 (15)	0.71332 (14)	0.0479 (3)	
H1	0.0021	0.3676	0.7320	0.058*	
C2	0.0765 (2)	0.16195 (16)	0.78664 (16)	0.0587 (4)	
H2	−0.0169	0.1499	0.8546	0.070*	
C3	0.2017 (2)	0.04936 (16)	0.76043 (17)	0.0612 (4)	
H3	0.1928	−0.0388	0.8094	0.073*	
C4	0.3409 (2)	0.06850 (15)	0.66055 (17)	0.0612 (4)	
H4	0.4267	−0.0072	0.6430	0.073*	
C5	0.35386 (18)	0.19854 (14)	0.58670 (14)	0.0504 (3)	
H5	0.4483	0.2100	0.5197	0.061*	
C6	0.22692 (15)	0.31286 (13)	0.61149 (12)	0.0397 (3)	
C7	0.23601 (14)	0.45452 (13)	0.53985 (12)	0.0382 (3)	
C8	0.21210 (16)	0.67461 (13)	0.49929 (14)	0.0445 (3)	
C9	0.30117 (16)	0.63430 (13)	0.38368 (13)	0.0432 (3)	
C10	0.37438 (15)	0.40978 (13)	0.30887 (12)	0.0382 (3)	
C11	0.53844 (15)	0.40107 (13)	0.23697 (12)	0.0407 (3)	
H11	0.6111	0.4461	0.2545	0.049*	
C12	0.59090 (15)	0.32340 (13)	0.13838 (12)	0.0411 (3)	
C13	0.48453 (16)	0.25187 (13)	0.11554 (12)	0.0426 (3)	
H13	0.5220	0.1981	0.0505	0.051*	
C14	0.32267 (16)	0.26149 (13)	0.19032 (13)	0.0423 (3)	
C15	0.26457 (15)	0.34325 (14)	0.28630 (13)	0.0422 (3)	
H15	0.1540	0.3527	0.3341	0.051*	
C16	0.1535 (2)	0.81699 (16)	0.52697 (18)	0.0652 (4)	
H16A	0.2035	0.8803	0.4532	0.098*	
H16B	0.1866	0.8185	0.6079	0.098*	
H16C	0.0327	0.8438	0.5376	0.098*	
C17	0.3660 (2)	0.71515 (16)	0.25083 (15)	0.0583 (4)	
H17A	0.3205	0.8118	0.2486	0.087*	
H17B	0.3325	0.6887	0.1816	0.087*	
H17C	0.4870	0.6973	0.2362	0.087*	
C18	0.85043 (19)	0.39811 (18)	0.05943 (17)	0.0611 (4)	
H18A	0.7898	0.4920	0.0379	0.092*	
H18B	0.9524	0.3835	−0.0063	0.092*	
H18C	0.8781	0.3800	0.1469	0.092*	
C19	0.2569 (3)	0.1152 (2)	0.0769 (2)	0.0763 (5)	
H19A	0.2843	0.1731	−0.0097	0.114*	
H19B	0.1653	0.0749	0.0777	0.114*	
H19C	0.3538	0.0433	0.0946	0.114*	
N1	0.17293 (13)	0.56251 (11)	0.59607 (11)	0.0428 (3)	
N2	0.31654 (13)	0.49237 (11)	0.40949 (10)	0.0390 (2)	
O1	0.74810 (12)	0.30746 (11)	0.05912 (10)	0.0557 (3)	
O2	0.20886 (13)	0.19468 (12)	0.17648 (11)	0.0618 (3)	

### Atomic displacement parameters (Å^2^)

**Table:**

	*U*^11^	*U*^22^	*U*^33^	*U*^12^	*U*^13^	*U*^23^
C1	0.0455 (7)	0.0507 (8)	0.0442 (7)	−0.0079 (6)	−0.0041 (5)	−0.0107 (6)
C2	0.0587 (9)	0.0609 (9)	0.0518 (8)	−0.0199 (7)	−0.0041 (7)	−0.0024 (7)
C3	0.0789 (11)	0.0466 (8)	0.0578 (9)	−0.0183 (7)	−0.0165 (8)	−0.0018 (7)
C4	0.0733 (10)	0.0452 (8)	0.0600 (9)	0.0014 (7)	−0.0140 (8)	−0.0151 (7)
C5	0.0511 (8)	0.0486 (8)	0.0470 (7)	−0.0037 (6)	−0.0042 (6)	−0.0141 (6)
C6	0.0422 (6)	0.0438 (7)	0.0358 (6)	−0.0080 (5)	−0.0086 (5)	−0.0128 (5)
C7	0.0346 (6)	0.0447 (7)	0.0367 (6)	−0.0069 (5)	−0.0038 (4)	−0.0154 (5)
C8	0.0441 (7)	0.0427 (7)	0.0487 (7)	−0.0083 (5)	−0.0071 (5)	−0.0157 (6)
C9	0.0442 (7)	0.0433 (7)	0.0444 (7)	−0.0108 (5)	−0.0088 (5)	−0.0111 (5)
C10	0.0394 (6)	0.0423 (6)	0.0332 (6)	−0.0063 (5)	−0.0055 (5)	−0.0123 (5)
C11	0.0385 (6)	0.0472 (7)	0.0383 (6)	−0.0115 (5)	−0.0052 (5)	−0.0122 (5)
C12	0.0382 (6)	0.0464 (7)	0.0352 (6)	−0.0071 (5)	−0.0012 (5)	−0.0098 (5)
C13	0.0477 (7)	0.0447 (7)	0.0359 (6)	−0.0084 (5)	−0.0040 (5)	−0.0145 (5)
C14	0.0438 (7)	0.0469 (7)	0.0403 (6)	−0.0121 (5)	−0.0098 (5)	−0.0119 (5)
C15	0.0362 (6)	0.0507 (7)	0.0403 (6)	−0.0094 (5)	−0.0033 (5)	−0.0144 (5)
C16	0.0730 (10)	0.0463 (8)	0.0749 (11)	−0.0117 (7)	0.0002 (8)	−0.0246 (8)
C17	0.0707 (10)	0.0539 (8)	0.0492 (8)	−0.0213 (7)	−0.0061 (7)	−0.0058 (6)
C18	0.0447 (8)	0.0767 (10)	0.0622 (9)	−0.0230 (7)	0.0072 (6)	−0.0213 (8)
C19	0.0892 (13)	0.0834 (12)	0.0785 (12)	−0.0404 (10)	−0.0069 (10)	−0.0412 (10)
N1	0.0423 (6)	0.0448 (6)	0.0426 (6)	−0.0081 (4)	−0.0026 (4)	−0.0177 (5)
N2	0.0402 (5)	0.0421 (6)	0.0360 (5)	−0.0094 (4)	−0.0031 (4)	−0.0136 (4)
O1	0.0451 (5)	0.0676 (6)	0.0545 (6)	−0.0168 (4)	0.0107 (4)	−0.0274 (5)
O2	0.0558 (6)	0.0790 (7)	0.0669 (7)	−0.0278 (5)	−0.0044 (5)	−0.0364 (6)

### Geometric parameters (Å, º)

**Table:**

C1—C2	1.377 (2)	C11—H11	0.9300
C1—C6	1.3922 (19)	C12—O1	1.3656 (16)
C1—H1	0.9300	C12—C13	1.3895 (19)
C2—C3	1.374 (2)	C13—C14	1.3800 (19)
C2—H2	0.9300	C13—H13	0.9300
C3—C4	1.381 (2)	C14—O2	1.3653 (16)
C3—H3	0.9300	C14—C15	1.3898 (18)
C4—C5	1.377 (2)	C15—H15	0.9300
C4—H4	0.9300	C16—H16A	0.9600
C5—C6	1.3897 (19)	C16—H16B	0.9600
C5—H5	0.9300	C16—H16C	0.9600
C6—C7	1.4669 (19)	C17—H17A	0.9600
C7—N1	1.3147 (16)	C17—H17B	0.9600
C7—N2	1.3729 (16)	C17—H17C	0.9600
C8—C9	1.3587 (19)	C18—O1	1.4231 (19)
C8—N1	1.3768 (18)	C18—H18A	0.9600
C8—C16	1.493 (2)	C18—H18B	0.9600
C9—N2	1.3882 (18)	C18—H18C	0.9600
C9—C17	1.481 (2)	C19—O2	1.406 (2)
C10—C15	1.3746 (18)	C19—H19A	0.9600
C10—C11	1.3857 (18)	C19—H19B	0.9600
C10—N2	1.4346 (16)	C19—H19C	0.9600
C11—C12	1.3831 (18)		
			
C2—C1—C6	120.68 (13)	C12—C13—H13	120.3
C2—C1—H1	119.7	O2—C14—C13	123.93 (12)
C6—C1—H1	119.7	O2—C14—C15	115.28 (11)
C3—C2—C1	120.60 (15)	C13—C14—C15	120.79 (12)
C3—C2—H2	119.7	C10—C15—C14	118.41 (11)
C1—C2—H2	119.7	C10—C15—H15	120.8
C2—C3—C4	119.25 (14)	C14—C15—H15	120.8
C2—C3—H3	120.4	C8—C16—H16A	109.5
C4—C3—H3	120.4	C8—C16—H16B	109.5
C5—C4—C3	120.64 (14)	H16A—C16—H16B	109.5
C5—C4—H4	119.7	C8—C16—H16C	109.5
C3—C4—H4	119.7	H16A—C16—H16C	109.5
C4—C5—C6	120.51 (14)	H16B—C16—H16C	109.5
C4—C5—H5	119.7	C9—C17—H17A	109.5
C6—C5—H5	119.7	C9—C17—H17B	109.5
C5—C6—C1	118.31 (13)	H17A—C17—H17B	109.5
C5—C6—C7	123.16 (12)	C9—C17—H17C	109.5
C1—C6—C7	118.47 (11)	H17A—C17—H17C	109.5
N1—C7—N2	110.87 (11)	H17B—C17—H17C	109.5
N1—C7—C6	123.45 (11)	O1—C18—H18A	109.5
N2—C7—C6	125.62 (11)	O1—C18—H18B	109.5
C9—C8—N1	110.47 (12)	H18A—C18—H18B	109.5
C9—C8—C16	128.66 (14)	O1—C18—H18C	109.5
N1—C8—C16	120.84 (13)	H18A—C18—H18C	109.5
C8—C9—N2	105.57 (12)	H18B—C18—H18C	109.5
C8—C9—C17	131.20 (13)	O2—C19—H19A	109.5
N2—C9—C17	123.15 (12)	O2—C19—H19B	109.5
C15—C10—C11	122.36 (12)	H19A—C19—H19B	109.5
C15—C10—N2	118.84 (11)	O2—C19—H19C	109.5
C11—C10—N2	118.80 (11)	H19A—C19—H19C	109.5
C12—C11—C10	118.02 (11)	H19B—C19—H19C	109.5
C12—C11—H11	121.0	C7—N1—C8	106.17 (11)
C10—C11—H11	121.0	C7—N2—C9	106.91 (10)
O1—C12—C11	124.12 (12)	C7—N2—C10	127.24 (11)
O1—C12—C13	114.83 (12)	C9—N2—C10	124.65 (11)
C11—C12—C13	121.03 (11)	C12—O1—C18	117.10 (11)
C14—C13—C12	119.32 (12)	C14—O2—C19	118.21 (12)
C14—C13—H13	120.3		
			
C6—C1—C2—C3	−0.2 (2)	C11—C10—C15—C14	1.80 (19)
C1—C2—C3—C4	0.8 (3)	N2—C10—C15—C14	−178.86 (11)
C2—C3—C4—C5	−0.7 (3)	O2—C14—C15—C10	177.80 (11)
C3—C4—C5—C6	0.0 (2)	C13—C14—C15—C10	−2.6 (2)
C4—C5—C6—C1	0.6 (2)	N2—C7—N1—C8	0.50 (13)
C4—C5—C6—C7	177.69 (13)	C6—C7—N1—C8	177.69 (11)
C2—C1—C6—C5	−0.5 (2)	C9—C8—N1—C7	−0.61 (15)
C2—C1—C6—C7	−177.72 (12)	C16—C8—N1—C7	177.60 (13)
C5—C6—C7—N1	−145.49 (13)	N1—C7—N2—C9	−0.22 (13)
C1—C6—C7—N1	31.57 (18)	C6—C7—N2—C9	−177.33 (11)
C5—C6—C7—N2	31.27 (19)	N1—C7—N2—C10	−168.08 (11)
C1—C6—C7—N2	−151.66 (12)	C6—C7—N2—C10	14.80 (19)
N1—C8—C9—N2	0.47 (15)	C8—C9—N2—C7	−0.16 (13)
C16—C8—C9—N2	−177.56 (14)	C17—C9—N2—C7	−177.29 (12)
N1—C8—C9—C17	177.28 (14)	C8—C9—N2—C10	168.10 (11)
C16—C8—C9—C17	−0.8 (3)	C17—C9—N2—C10	−9.03 (19)
C15—C10—C11—C12	0.70 (19)	C15—C10—N2—C7	50.18 (17)
N2—C10—C11—C12	−178.64 (11)	C11—C10—N2—C7	−130.45 (13)
C10—C11—C12—O1	179.18 (12)	C15—C10—N2—C9	−115.66 (14)
C10—C11—C12—C13	−2.44 (19)	C11—C10—N2—C9	63.70 (16)
O1—C12—C13—C14	−179.85 (12)	C11—C12—O1—C18	−13.3 (2)
C11—C12—C13—C14	1.62 (19)	C13—C12—O1—C18	168.19 (13)
C12—C13—C14—O2	−179.50 (12)	C13—C14—O2—C19	−2.4 (2)
C12—C13—C14—C15	1.0 (2)	C15—C14—O2—C19	177.17 (14)

### Hydrogen-bond geometry (Å, º)

**Table:**

*D*—H···*A*	*D*—H	H···*A*	*D*···*A*	*D*—H···*A*
C15—H15···N1^i^	0.93	2.62	3.516 (2)	162

Symmetry code: (i) −*x*, −*y*+1, −*z*+1.
